# LncRNA ZEB1-AS1: Um Novo Ator no Controle Epigenético da Hipertrofia Cardíaca

**DOI:** 10.36660/abc.20250692

**Published:** 2025-12-11

**Authors:** Fernando R. Giugni

**Affiliations:** 1 Hospital das Clínicas da Faculdade de Medicina da Universidade de São Paulo Instituto do Coração Laboratório de Genética e Cardiologia Molecular São Paulo SP Brasil Laboratório de Genética e Cardiologia Molecular, Instituto do Coração do Hospital das Clínicas da Faculdade de Medicina da Universidade de São Paulo, São Paulo, SP – Brasil; 2 University of Texas Southwestern Medical Center Division of Cardiology Dallas TX EUA Division of Cardiology, University of Texas Southwestern Medical Center, Dallas, TX – EUA

**Keywords:** Hipertrofia, RNA Longo não Codificante

A hipertrofia cardíaca representa uma resposta adaptativa fundamental do miocárdio ao estresse hemodinâmico.^1^ Embora inicialmente compensatória, a hipertrofia persistente predispõe à remodelação maladaptativa, disfunção diastólica e, por fim, à insuficiência cardíaca. Apesar do progresso substancial no bloqueio neuro-hormonal e no controle da pressão arterial, as terapias que visam diretamente os mecanismos moleculares que sustentam a hipertrofia patológica permanecem limitadas. Nos últimos anos, a atenção crescente tem se voltado para os RNAs não codificantes — particularmente os RNAs não codificantes longos (lncRNAs) — como reguladores do crescimento, da contratilidade e da sobrevivência dos cardiomiócitos.^2,3^ Compreender como essas moléculas orquestram a expressão gênica e a remodelação da cromatina oferece novas oportunidades para modular o processo hipertrófico em seu núcleo regulador.

Embora vários lncRNAs, como TUG1,^4^ NEAT1,^5^ e PVT1^6^ tenham sido associados à resposta hipertrófica, a diversidade desses transcritos e a especificidade contextual de seus efeitos sugerem que muitos reguladores-chave permanecem desconhecidos. Entre eles, o ZEB1-AS1, originalmente caracterizado na biologia do câncer como um modulador da proliferação e migração celular, não havia sido estudado no coração.^7^ Ainda não se sabe se esse lncRNA contribui para a remodelação cardíaca e por meio de quais intermediários moleculares.

Nesta edição, Cao et al.^8^ abordam essa lacuna de conhecimento fornecendo evidências de que ZEB1-AS1 atua como um regulador pró-hipertrófico por meio de um eixo de sinalização ZEB1-AS1/miR-186-5p/HDAC2 anteriormente não reconhecido.^8^ Os pesquisadores combinaram a análise de tecido cardíaco humano com estudos mecanísticos em cardiomiócitos cultivados para elucidar como essa via contribui para o crescimento hipertrófico. Eles primeiro demonstraram que a expressão de ZEB1-AS1 é aumentada no tecido miocárdico de pacientes com hipertrofia ventricular esquerda em comparação com corações de doadores normais. Para estender essas descobertas em um sistema experimental, eles usaram cardiomiócitos AC16 humanos cultivados tratados com isoproterenol (ISO) para induzir um fenótipo hipertrófico. Neste modelo, a estimulação ISO recapitulou características cardinais da hipertrofia — área de superfície celular aumentada e expressão elevada de ANP, BNP e β-MHC — e foi acompanhada por um aumento paralelo nos níveis de ZEB1-AS1.

O silenciamento de ZEB1-AS1 atenuou significativamente a hipertrofia induzida por ISO, reduzindo tanto o aumento celular quanto a expressão de marcadores hipertróficos. Experimentos mecanísticos revelaram então que ZEB1-AS1 funciona como uma esponja molecular para miR-186-5p, um microRNA previamente implicado na aterosclerose. Ensaios de ligação e estudos de repórter de luciferase dupla confirmaram a interação direta entre ZEB1-AS1 e miR-186-5p, bem como entre miR-186-5p e histona desacetilase 2 (HDAC2). Por meio desse mecanismo competitivo de RNA endógeno (ceRNA), ZEB1-AS1 alivia a repressão de HDAC2 mediada por miR-186-5p, levando ao aumento da expressão de HDAC2 e consequente ativação da sinalização hipertrófica. Experimentos de resgate corroboraram este modelo: a inibição de miR-186-5p reverteu os efeitos protetores da redução de ZEB1-AS1, restaurando a expressão do marcador hipertrófico.

Esses dados delineiam uma cascata regulatória — a regulação positiva de ZEB1-AS1 promove hipertrofia cardíaca ao suprimir o miR-186-5p, aumentando assim a atividade da HDAC2. Os resultados ampliam observações anteriores que ligavam a HDAC2 à remodelação da cromatina e ao controle transcricional no coração hipertrófico,^9-12^ posicionando agora um lncRNA como um regulador epigenético. A demonstração de que a expressão da HDAC2 pode ser ajustada por meio de um mecanismo baseado em RNA sugere novas possibilidades para direcionar reguladores a montante em vez da enzima em si — uma estratégia potencialmente mais seletiva para intervenção terapêutica.

Vários aspectos do trabalho merecem destaque. O uso de amostras de miocárdio humano e ensaios funcionais in vitro reforça a plausibilidade biológica e a relevância translacional. O estudo também integra múltiplas técnicas complementares — qRT-PCR, *Western blotting*, ensaios de repórter de luciferase e imunoprecipitação de RNA — para mapear interações moleculares. Juntas, essas abordagens fornecem uma narrativa mecanicista coerente que vincula a desregulação transcriptômica à remodelação fenotípica.

No entanto, limitações importantes moderam a interpretação. O tamanho da amostra de espécimes de tecido humano é modesto, e o estudo carece de validação funcional in vivo em modelos animais de sobrecarga de pressão ou ativação neuro-hormonal. Ainda não se sabe se a expressão de ZEB1-AS1 muda dinamicamente durante a transição da hipertrofia compensada para a insuficiência cardíaca. Além disso, como os lncRNAs frequentemente exercem efeitos pleiotrópicos em múltiplos tipos de células, a especificidade de ZEB1-AS1 para cardiomiócitos deve ser confirmada em estudos futuros. Finalmente, embora os cardiomiócitos tratados com ISO forneçam um modelo conveniente, eles capturam apenas um subconjunto dos complexos estímulos mecânicos e metabólicos que impulsionam a hipertrofia in vivo.

Apesar dessas ressalvas, o estudo de Cao et al.^8^ expande nossa compreensão de como o genoma não codificante contribui para a remodelação estrutural do coração. Ele posiciona o ZEB1-AS1 como um potencial alvo terapêutico para hipertrofia cardíaca. De uma perspectiva translacional, estratégias voltadas para a inibição do ZEB1-AS1 — usando oligonucleotídeos *antisense*, siRNA ou ferramentas baseadas em CRISPR — poderiam potencialmente atenuar a remodelação mediada por HDAC2, poupando funções essenciais da cromatina reguladas por outras isoformas de HDAC. À medida que o campo avança em direção à terapêutica direcionada por RNA e à modulação de precisão da expressão gênica, estudos como este ressaltam como a decodificação do transcriptoma não codificante pode revelar novos caminhos para combater a remodelação cardíaca maladaptativa e a progressão da insuficiência cardíaca.
