# Supplementary material for: LncRNA ZEB1-AS1: Um Novo Ator no Controle Epigenético da Hipertrofia Cardíaca
Source: Arq Bras Cardiol. 2025 Dec 11;122(11):e20250692. [Article in Portuguese] doi: 10.36660/abc.20250692 (PMC12978280; doi:10.36660/abc.20250692)
Supplement: Translation

# Translation: LncRNA ZEB1-AS1: A New Player in Epigenetic Control of Cardiac Hypertrophy

**DOI:** 10.36660/abc.20250692i

**Authors:** Fernando R. Giugni

**Affiliations:** 1 São Paulo SP Brazil Laboratório de Genética e Cardiologia Molecular, Instituto do Coração do Hospital das Clínicas da Faculdade de Medicina da Universidade de São Paulo, São Paulo, SP – Brazil; 2 Dallas TX USA Division of Cardiology, University of Texas Southwestern Medical Center, Dallas, TX – USA

**Keywords:** Hypertrophy, Long Noncoding RNA

Cardiac hypertrophy represents a fundamental adaptive response of the myocardium to hemodynamic stress.^1^ Although initially compensatory, persistent hypertrophy predisposes to maladaptive remodeling, diastolic dysfunction, and ultimately heart failure. Despite substantial progress in neurohormonal blockade and blood pressure control, therapies directly targeting the molecular mechanisms that sustain pathological hypertrophy remain limited. In recent years, growing attention has turned toward noncoding RNAs—particularly long noncoding RNAs (lncRNAs)—as regulators of cardiomyocyte growth, contractility, and survival.^2,3^ Understanding how these molecules orchestrate gene expression and chromatin remodeling offers new opportunities to modulate the hypertrophic process at its regulatory core.

While several lncRNAs such as TUG1,^4^ NEAT1,^5^ and PVT1^6^ have been linked to the hypertrophic response, the diversity of these transcripts and the context-specificity of their effects suggest that many key regulators remain unidentified. Among these, ZEB1-AS1, originally characterized in cancer biology as a modulator of cell proliferation and migration, had not been studied in the heart.^7^ Whether this lncRNA contributes to cardiac remodeling, and through which molecular intermediates, remains unknown.

In this issue, Cao et al.^8^ address this knowledge gap by providing evidence that ZEB1-AS1 acts as a pro-hypertrophic regulator through a previously unrecognized ZEB1-AS1/miR-186-5p/HDAC2 signaling axis.^8^ The investigators combined analysis of human heart tissue with mechanistic studies in cultured cardiomyocytes to elucidate how this pathway contributes to hypertrophic growth. They first demonstrated that ZEB1-AS1 expression is increased in myocardial tissue from patients with left ventricular hypertrophy compared with normal donor hearts. To extend these findings in an experimental system, they used cultured human AC16 cardiomyocytes treated with isoproterenol (ISO) to induce a hypertrophic phenotype. In this model, ISO stimulation recapitulated cardinal features of hypertrophy—enlarged cell surface area and elevated expression of ANP, BNP, and β-MHC—and was accompanied by a parallel increase in ZEB1-AS1 levels.

Silencing ZEB1-AS1 significantly attenuated ISO-induced hypertrophy, reducing both cellular enlargement and expression of hypertrophic markers. Mechanistic experiments then revealed that ZEB1-AS1 functions as a molecular sponge for miR-186-5p, a microRNA previously implicated in atherosclerosis.^9-12^ Binding assays and dual-luciferase reporter studies confirmed the direct interaction between ZEB1-AS1 and miR-186-5p, as well as between miR-186-5p and histone deacetylase 2 (HDAC2). Through this competitive endogenous RNA (ceRNA) mechanism, ZEB1-AS1 relieves miR-186-5p-mediated repression of HDAC2, leading to increased HDAC2 expression and consequent activation of hypertrophic signaling. Rescue experiments supported this model: inhibition of miR-186-5p reversed the protective effects of ZEB1-AS1 knockdown, restoring hypertrophic marker expression.

These data delineate a regulatory cascade—ZEB1-AS1 upregulation promotes cardiac hypertrophy by suppressing miR-186-5p, thereby enhancing HDAC2 activity. The results extend prior observations linking HDAC2 to chromatin remodeling and transcriptional control in the hypertrophic heart,^10-12^ now positioning an lncRNA as an epigenetic regulator. Demonstrating that HDAC2 expression can be tuned through an RNA-based mechanism suggests new possibilities for targeting upstream regulators rather than the enzyme itself—a potentially more selective strategy for therapeutic intervention.

Several aspects of the work deserve emphasis. The use of both human myocardial samples and in vitro functional assays strengthens biological plausibility and translational relevance. The study also integrates multiple complementary techniques—qRT-PCR, Western blotting, luciferase reporter assays, and RNA immunoprecipitation—to map molecular interactions. Together, these approaches provide a coherent mechanistic narrative linking transcriptomic dysregulation to phenotypic remodeling.

Nevertheless, important limitations temper interpretation. The sample size of human tissue specimens is modest, and the study lacks in vivo functional validation in animal models of pressure overload or neurohormonal activation. Whether ZEB1-AS1 expression changes dynamically during the transition from compensated hypertrophy to heart failure remains unknown. Furthermore, as lncRNAs often exert pleiotropic effects across multiple cell types, the cardiomyocyte specificity of ZEB1-AS1 should be confirmed in future studies. Finally, while ISO-treated cardiomyocytes provide a convenient model, they capture only a subset of the complex mechanical and metabolic stimuli driving hypertrophy in vivo.

Despite these caveats, the study by Cao et al.^8^ expands our understanding of how the noncoding genome contributes to structural remodeling of the heart. It positions ZEB1-AS1 as a potential therapeutic target for cardiac hypertrophy. From a translational perspective, strategies aimed at inhibiting ZEB1-AS1—using antisense oligonucleotides, siRNA, or CRISPR-based tools—could potentially attenuate HDAC2-mediated remodeling while sparing essential chromatin functions regulated by other HDAC isoforms. As the field moves toward RNA-directed therapeutics and precision modulation of gene expression, studies like this underscore how decoding the noncoding transcriptome may reveal new avenues to counter maladaptive cardiac remodeling and heart failure progression.
